# The associations between the credibility of the tobacco control regulatory body and smoking behavior change among Saudi smokers

**DOI:** 10.18332/tid/155814

**Published:** 2022-12-02

**Authors:** Abdullah M. Alanazi, Sarah S. Monshi, Nada A. Alfahadi, Sadeem S. Alsayari, Foton S. Alkhonain, Norah M. Alsulami, Tareq F. Alotaibi, Saleh S. Algarni, Hassan Y. Abunurah, Abdulmohsen H. Al-Zalabani, Taha T. Ismaeil

**Affiliations:** 1 Department of Respiratory Therapy, College of Applied Medical Sciences, King Saud bin Abdulaziz University for Health Sciences, Riyadh, Saudi Arabia; 2 King Abdullah International Medical Research Center, Riyadh, Saudi Arabia; 3 Department of Health Services Management, College of Public Health and Health Informatics, Umm Al-Qura University, Mecca, Saudi Arabia; 4 Department of Family and Community Medicine, College of Medicine, Taibah University, Medina, Saudi Arabia

**Keywords:** communication, policy, Saudi Arabia, smokers, smoking cessation

## Abstract

**INTRODUCTION:**

Recently, Saudi Arabia has extensively reformed its tobacco control policies and extended its smoking cessation services. A public outrage on social media among smokers was witnessed, especially after the implementation of plain cigarette packaging, which might have discredited the significant efforts of tobacco treatment services and tobacco control policies. However, it is not known how the credibility of the tobacco control regulatory body among Saudi smokers might affect their smoking behavior.

**METHODS:**

Saudi tobacco smokers (n=511) were recruited using a convenience sampling technique. A cross-sectional survey was conducted comprising questions related to the credibility of the tobacco control regulatory body (modified Food and Drug Administration Tobacco Credibility Scale), quit attempts, use of nicotine replacement therapy (NRT), and motivation to change smoking behavior in the future. Logistic and linear regression models were used for the analysis.

**RESULTS:**

The public interest subscale of the credibility of the tobacco control regulatory body, was positively associated with confidence in changing smoking behavior (β=0.204; 95% CI: 0.078–0.713; t=2.449, p=0.015) and readiness to change smoking behavior (β=0.237; 95% CI: 0.127–0.727; t=2.802, p=0.005). Moreover, the subscale of expertise was positively associated with confidence in changing smoking behavior (β=0.190; 95% CI: 0.006–0.697; t=1.999, p=0.046) and readiness to change smoking behavior (β=0.225; 95% CI: 0.063–0.710; t=2.352, p=0.019). However, public interest in the credibility of the tobacco control regulatory body was negatively associated with NRT use among smokers who tried to quit (adjusted odds ratio, AOR=0.691; 95% CI: 0.526–0.909). The credibility of the tobacco control regulatory body, however, was not associated with the last month’s or ever quit attempts.

**CONCLUSIONS:**

The credibility of the tobacco control regulatory body was positively associated with motivation to change smoking behavior but negatively associated with NRT use. Optimizing communication tools with the public is a potential avenue for improving smoking treatment and prevention in Saudi Arabia.

## INTRODUCTION

Tobacco smoking causes several chronic diseases, including cancers, lung diseases, and cardiovascular diseases^1-3^. Similar to several countries located in the Eastern Mediterranean region, Saudi Arabia (SA) is witnessing a high prevalence of tobacco use among adults (19.8%)^4,5^. Consequently, the Saudi government has launched several tobacco control initiatives to combat tobacco smoking^6^. In 2015, an anti-smoking law was enacted in SA to tackle the issue of tobacco use at the national level^7^. The law is based on the World Health Organization Framework Convention on Tobacco Control (FCTC) treaty that aims to help nations address the issue of tobacco use. The law includes different provisions that ensure the reduction of demand and supply for tobacco use, such as imposing smoke-free policies in public places, prohibiting the sale of tobacco to minors, and regulating tobacco content, packaging, and labeling. A further step was taken by the Saudi government when it imposed 100% taxation on tobacco products in 2017 and adopted plain cigarette packaging^6,7^. All of these policies were implemented to prevent and decrease tobacco smoking in the nation^8,9^.

The tobacco industry is known to manipulate the public to react against implementing the FCTC treaty by promoting deceptive information about the fallacy of FCTC effectiveness^10,11^. For example, when the Australian government implemented plain packaged cigarettes in 2012, the public expressed their dissatisfaction with the policy and the quality of the cigarettes^11^. This alerted policy makers to take aggressive steps against any interventions from the tobacco industry^12,13^. For example, in Nigeria, strong efforts from tobacco advocates and tobacco regulatory groups have tried to gain public support and pushed for the tobacco industry by manipulating and discrediting tobacco control policies^12,13^.

Public support is a fundamental component of tobacco control initiatives. Smokers may exhibit defensive behaviors in denying anti-smoking rules. They may internalize stigma related to smoking by continuing smoking and presenting rebellious attitudes toward tobacco control policies and smoking cessation services^14,15^. In SA, the public has had a noticeable response to tobacco control interventions^6^. An observable example was the implementation of plain packaged cigarettes in the country as smokers reacted on social media platforms complaining of the quality and taste of the new cigarettes^6^. On the other hand, the official response from the tobacco control regulatory body was not satisfactory to the smokers in addressing their concerns about the new cigarettes^6^.

The tobacco control regulatory body in SA comprises official representatives from the Food and Drug Authority and 10 Ministries: Health; Interior Affairs; Municipal and Rural Affairs; Finance; Media; Education; Commerce; Human Resources and Social Development; Sport, and Islamic Affairs. These representatives are governed by the umbrella of the National Tobacco Control Committee with two primary goals: protecting members of the society from tobacco use, and reducing the prevalence of tobacco use, especially among young individuals. Each agency is responsible for operationalizing the National Tobacco Control Committee resolutions, such as imposing tobacco-free places, promoting tobacco prevention campaigns, and implementing plain packaging for cigarettes according to their capacity^16^.

A recent case study of SA, in implementing plain packaged cigarettes that documented public response against the policy, demonstrates the lack of effective communication between the tobacco control regulatory body and smokers before and during the implementation of tobacco control policies to facilitate the implementation process^6^. Ideally, reciprocal credibility and common grounds of trust should be sought from smokers and tobacco control regulatory bodies to increase the effectiveness of tobacco control and smoking cessation services^17,18^. For instance, it was observed in the US that smokers who have positive views about the Food and Drug Administration (FDA) held views of greater credibility for the FDA as a regulatory body and a more negative view of the tobacco industry^19^. Such attitudes toward the FDA and tobacco industry could affect smokers’ behavior towards quitting and seeking treatment^20,21^. However, in the context of SA, it is unclear what the status of the credibility of the tobacco control regulatory body is and how it may predict smoking behavior change.

SA has a progressive agenda for improving the quality of life and curbing non-communicable diseases^22^. Implementing tobacco control policies by the tobacco control regulatory body aimed at preventing the initiation of tobacco use and assisting smokers to quit smoking is an example of its vision. Therefore, this study aimed to assess how the credibility of the tobacco control regulatory body might affect smoking behavior change in terms of the motivation to change smoking behavior, quit attempts, and the use of nicotine replacement therapy (NRT), among Saudi smokers. Specifically, we hypothesized that a higher credibility of tobacco control regulatory body among smokers would predict greater motivation to change smoking behavior, greater quit attempts, and greater NRT use.

## METHODS

### Participants

This study used a cross-sectional survey design. A convenience sample of 511 Saudi adults aged ≥18 years who smoked tobacco was recruited through word of mouth. The individuals were considered tobacco smokers if they had smoked tobacco cigarettes and/or tobacco hookah (even one puff) in the last 30 days^23,24^. An electronic self-administered questionnaire comprising informed consent and closed-ended questions was used to collect the data. For the study questions that were adapted from previous studies and published scales^25-27^, the questions were translated from English to Arabic in two steps. First, forward-backward translations of experts in linguistics were performed to ensure the accuracy of item content. Second, cultural adaptation of the questions was used to pilot test the questionnaire by reviewing it with Saudi adults who smoke tobacco to assess the clarity of each question. After the pilot test, no questions from the original scale were excluded or modified. The study was conducted from June 2021 to August 2021.

### Measures

*Sociodemographic characteristics*

Sociodemographic characteristics were collected from participants including age, sex (female or male), social status (single, married, or divorced/widowed), occupational status (having a job, not having a job, or a student), and education level (less than high school, high school, diploma, Bachelor’s degree, Master’s degree/medical board, or PhD/medical sub-specialty).

*Smoking behavior change*

To assess changes in smoking behavior among Saudi smokers, we used different items that reflect the degree to which a smoker might change his/her behavior. Items included motivation to change, last month’s quit attempts, ever quit attempts, and NRT use. The Motivation to Change Scale (MCQ) was used to assess the motivation to change smoking behavior^25,26^. The MCQ is a 3-item questionnaire that measures the motivation to change substance use behavior that was found to be valid for smoking behavior^25,26^. The MCQ version of the measure specific to smoking behavior was used by asking: ‘How important is it to you to make changes in your smoking behavior?’ denoted as the importance of change; ‘How confident are you that you would be able to make changes in your smoking behavior if you decided to do so?’ denoted as the confidence to change; and ‘How ready are you to make changes in your smoking behavior?’ denoted as readiness to change. Each item was examined separately on a scale 0–10, with a higher score representing greater motivation to change smoking behavior.

In addition, three questions were asked to assess last month’s quit attempts, ever quit attempts, and NRT use. Last month’s quit attempts were measured by asking: ‘Have you ever tried to quit tobacco smoking in the last month?’. Ever quit attempts were measured by asking: ‘Have you ever tried to quit tobacco smoking in the past?’. NRT use was measured by asking: ‘Have you used NRT (this includes nicotine patches and nicotine gum) to quit?’. Each question was assessed using a binary indicator (no=0 and yes=1).

*Credibility of the tobacco control regulatory body*

To measure the credibility of the tobacco control regulatory body, a modified version of the USFDA Tobacco Credibility Scale (FDA-TCS) was adopted. The FDA-TCS is a valid and reliable instrument (Cronbach’s alpha=0.95)^27^. It monitors the credibility of tobacco control organizations. The instrument comprises three subscales: 1) public interest, which assesses the degree to which the tobacco control regulatory body acts in the interests of the public; 2) trust, which measures the extent to which the tobacco control regulatory body is believed to present correct information related to tobacco; and 3) expertise, which examines the extent to which the tobacco control regulatory body knows the correct information about tobacco. The final FDA-TCS instrument comprised 17 items: 1) public interest with six items, 2) trust with six items, and 3) expertise with five items. Responses to each item were developed on a 7-point scale ranging from strongly disagree (1) to strongly agree (7). For this study, we modified the FDA-TCS instrument to suit the context of tobacco control in SA by replacing ‘FDA’ with ‘the tobacco control regulatory body in SA’ in every relevant item of the instrument. This modification was made because the tobacco control regulatory body in SA is represented by numerous ministries, as described earlier. The final score for the credibility of the tobacco control regulatory body was computed by the mean of each subscale for each participant (1=low and 7=high). A higher mean score indicated greater credibility of the tobacco control regulatory body in SA among smokers.

### Statistical analysis

Descriptive statistics of percentages and means were reported to represent participant characteristics in the univariate analysis. Bivariate analysis was conducted to reveal the credibility of the tobacco control regulatory body across participant characteristics by conducting independent t-tests and analysis of variance tests. Bivariate analyses of independent t-tests and chi-squared tests were conducted to assess the differences between females and males across the study’s primary variables. Finally, two statistical tests were performed to conduct multivariate models. First, a linear regression was conducted to examine the associations between the credibility of the tobacco control regulatory body and motivation to change smoking behavior. Second, logistic regression was used to examine the associations between the credibility of the tobacco control regulatory body and last month’s quit attempts, ever quit attempts, and use of NRT. All models were controlled for demographic characteristics (age, sex, social status, occupational status, and education level). Missing values from the demographic characteristics were considered missing completely at random. SPSS version 26 was used for analysis. Statistical significance was set at p<0.05.

## RESULTS

Most of the participants were aged between 18 and 24 years and were mostly males, single, and employed. Moreover, 39.8% and 31.8% of the participants had a Bachelor’s degree and had completed high school education, respectively. Bivariate analysis showed no significant differences in the credibility of the tobacco control regulatory body across the sociodemographic characteristics of smokers (Table 1). Table 2 reveals that according to the scale of the motivation to change smoking behavior, most of the participants reported that changing smoking behavior was important (mean=6.6, SD=3.3), they were confident to make changes (mean=6.2, SD=3.3), and they were ready to make changes (mean=6.5, SD=3.1). While only 22.0% of participants tried to quit tobacco smoking in the last month, 66.4% tried to quit smoking in the past. Only 16.1% of the participants used NRT to quit tobacco smoking. The participants’ mean score of the cumulative credibility of the tobacco control regulatory body was in the middle (mean=4.4, SD=1.6) along with its subscales that included public interest (mean=4.3, SD=1.7), trust (mean=4.3, SD=1.9), and expertise (mean=4.7, SD=1.7). Women were less motivated to change their smoking behavior (mean=5.7, SD=3.0) and reported fewer quit attempts (34.0%) and NRT use (5.2%) than men (mean=6.6, SD=2.8, 76.3%, 19.3%, respectively).

**Table 1:** The mean score of cumulative credibility score across the sample characteristics of Saudi smokers, 2021

*Variable*	*n (%)*	*Score of cumulative credibility Mean ± SD*	*Test statistic*	*p*
**Age** (years)			1.63	0.137
18–24	205 (42.4)	4.58 ± 1.63		
25–29	52 (10.8)	4.22 ± 1.76		
30–34	25 (5.2)	4.52 ± 1.84		
35–39	43 (8.9)	4.47 ± 1.71		
40–44	36 (7.5)	5.07 ± 1.93		
45–49	47 (9.7)	4.59 ± 1.60		
≥50	75 (15.5)	4.05 ± 1.52		
**Sex**			1.66	0.097
Female	133 (26.0)	4.69 ± 1.68		
Male	378 (74.0)	4.36 ± 1.67		
**Social status**			0.25	0.777
Single	262 (50.8)	4.49 ± 1.64		
Married	244 (47.3)	4.40 ± 1.72		
Divorced/widowed	10 (2.0)	4.19 ± 1.76		
**Occupational status**			1.30	0.272
Employed	263 (51.1)	4.32 ± 1.72		
Unemployed	70 (13.6)	4.59 ± 1.71		
Student	182 (35.3)	4.59 ± 1.59		
**Education level**			2.14	0.060
Less than high school	20 (3.9)	4.72 ± 2.10		
High school	164 (31.8)	4.75 ± 1.58		
Diploma	63 (12.2)	4.13 ± 1.70		
Bachelor’s	205 (39.8)	4.41 ± 1.67		
Master’s/medical board	53 (10.3)	4.10 ± 1.77		
PhD/medical sub-specialty	10 (1.9)	3.65 ± 1.44		

**Table 2:** The univariate and bivariate analyses of smoking behavior change scales and credibility of tobacco control regulatory body with differences in sex (N=511)

	*All (n=511) Mean ± SD or n (%)*	*Female (n=133) Mean ± SD or n (%)*	*Male (n=378) Mean ± SD or n (%)*	*Test statistic*	*p*
**Motivation to change smoking behavior**					
Importance of change	6.61 ± 3.32	5.72 ± 3.44	6.81 ± 3.27	2.55	0.011
Confidence to change	6.29 ± 3.34	5.64 ± 3.57	6.44 ± 3.27	1.85	0.065
Readiness to change	6.55 ± 3.15	5.99 ± 3.32	6.68 ± 3.10	1.69	0.091
Cumulative score	6.46 ± 2.90	5.73 ± 3.02	6.63 ± 2.85	2.40	0.017
**Last-month quit attempt**				0.37	0.539
No	350 (78.0)	78 (75.7)	268 (78.6)		
Yes	99 (22.0)	25 (24.3)	73 (21.4)		
**Ever quit attempt**				64.96	<0.001
No	152 (33.6)	70 (66.0)	81 (23.7)		
Yes	301 (66.4)	36 (34.0)	261 (76.3)		
**NRT use**				12.92	<0.001
No	396 (83.9)	109 (94.8)	284 (80.7)		
Yes	76 (16.1)	6 (5.2)	68 (19.3)		
**Credibility of tobacco control regulatory body**					
Public interest	4.32 ± 1.72	4.61 ± 1.63	4.23 ± 1.73	1.83	0.067
Trust	4.31 ± 1.93	4.45 ± 1.91	4.26 ± 1.94	0.76	0.442
Expertise	4.70 ± 1.79	4.91 ± 1.78	4.63 ± 1.79	1.28	0.200
Cumulative score	4.44 ± 1.68	4.69 ± 1.68	4.36 ± 1.67	1.66	0.097

Table 3 shows the associations between the credibility of the tobacco control regulatory body and changes in smoking behavior. In particular, the degree to which the tobacco control regulatory body acted in the interest of the public was significantly associated with greater confidence in changing smoking behavior (β=0.204; 95% CI: 0.078–0.713; t=2.449, p=0.015) and readiness to change behavior (β=0.237; 95% CI: 0.127–0.727; t=2.802, p=0.005). Similarly, the perception that the tobacco control regulatory body had expertise in tobacco control was positively associated with greater confidence in changing behavior (β=0.190; 95% CI: 0.006–0.697; t=1.999, p=0.046) and readiness to change behavior (β=0.225; 95% CI: 0.063–0.710; t=2.352, p=0.019). Interestingly, those who perceived that the tobacco control regulatory body acted in the interest of the public were less likely to use NRT (AOR=0.691; 95% CI: 0.526–0.909). Finally, trust in the tobacco control regulatory body was not associated with any changes in smoking behavior.

**Table 3:** The associations between the credibility of tobacco control policies with smoking behavior change items

*Smoking behavior change items (dependent variables)*	*Credibility of tobacco control regulatory body (independent variables)*								
	*Public interest*			*Trust*			*Expertise*		
*Motivation to change*	*β (95% CI)*	*t*	*p*	*β (95% CI)*	*t*	*p*	*β (95% CI)*	*t*	*p*
Importance of change	0.166 (-0.006–0.640)	1.932	0.054	0.006 (-0.335–0.355)	0.057	0.955	0.167 (-0.043–0.658)	1.735	0.085
Confidence to change	0.204 (0.078–0.713)	2.449	0.015*	0.034 (-0.281–0.398)	0.339	0.735	0.190 (0.006–0.697)	1.999	0.046*
Readiness to change	0.237 (0.127–0.727)	2.802	0.005*	-0.044 (-0.39–0.248)	-0.438	0.661	0.225 (0.063–0.710)	2.352	0.019*
***Quit status***	***AOR***	***95% CI***		***AOR***	***95% CI***		***AOR***	***95% CI***	
Last-month quit attempt	1.068	0.829–1.375		1.041	0.786–1.378		1.012	0.760–1.349	
Ever quit attempt	0.910	0.703–1.180		1.056	0.811–1.374		1.097	0.832–1.446	
NRT use	0.691*	0.526–0.909		1.022	0.763–1.370		1.206	0.904–1.610	

Controlled for age, sex, education level, occupational status, and social status. NRT: nicotine replacement therapy.

* Statistically significant at p<0.05.

## DISCUSSION

This study is the first to assess the credibility of the tobacco control regulatory body in Saudi smokers. The findings revealed novel associations between the credibility of the tobacco control regulatory body in SA and smoking behavior change, including the motivation to change smoking behavior, quit attempts, and NRT use. The study found that a greater degree to which smokers perceived that the tobacco control regulatory body acted in the public’s interest and had expertise in knowing the correct information about tobacco was associated with greater confidence and readiness to change smoking behavior. However, a greater degree to which smokers perceived that the tobacco control regulatory body acted in the public’s interest was associated with lower odds of NRT use among smokers who tried to quit.

Earlier research has indicated that the perceived credibility of a regulatory organization predicts health outcomes and behaviors^28^. Greater perceived credibility of policies might be an indicator of effective communication between the regulatory bodies and the public^29^. Additionally, a high credibility of tobacco control regulatory bodies increases compliance with tobacco control policies^27,28^. Similarly, the perceived credibility of tobacco control policies among Saudi smokers predicted confidence and readiness to change smoking behavior. As an increase in credibility may lead to higher compliance with tobacco control policies, opposition toward the implementation of future tobacco control policies in the nation would decrease. Such findings might be linked to the mediated effects of negative attitudes towards the tobacco industry, despite the attachment to tobacco use^20,21^. However, this claim should be investigated in future studies.

Interestingly, this study showed a negative association between the credibility of the tobacco control regulatory body and NRT use. The greater degree to which the tobacco control regulatory body acted in the public’s interest predicted lower odds of using NRT among smokers who attempted to quit. This adverse association might be due to physiological and cognitive differences such as nicotine dependence and outcome expectancies of NRT effectiveness^30-34^. Indeed, smokers may have positive expectations of the tobacco control regulatory body acting in the public’s interest but do not necessarily hold positive expectations about the effectiveness of using NRT to quit, especially among heavy smokers who are less likely to seek treatment and may quit using other aids^35^. Addressing the interaction effects of nicotine dependence and outcome expectancies of NRT effectiveness in the association between the credibility of the tobacco control regulatory body and NRT use might provide future intervention avenues.

This study’s findings stress communicating the appropriate channels of tobacco use treatment, including NRT.^6^ Smokers may quit using different aids^35^. For example, ‘cold turkey’, wherein smokers abruptly stop smoking without assistance, is not recommended as a treatment method^36^. Moreover, NRT could be misperceived as an ineffective treatment by smokers, besides NRT might be inadequately prescribed and offered by healthcare providers even though NRT is freely available to any smoker in the country^37^. In fact, NRT use has been found as the most effective aid to help smokers to quit^38^; therefore, policymakers may need to optimize health communication strategies to direct smokers to the appropriate treatment services, as well as to healthcare providers to support smoking cessation with NRT.

This study, however, has significant implications for stakeholders responsible for developing and implementing tobacco control policies and services. To strengthen the credibility of tobacco control, which was found to be associated with motivation to change smoking behavior, stakeholders may increase transparency, reveal their expertise, and engage the public in the decision-making process to increase their willingness to change risky health behaviors, including smoking behavior. Engaging communities such as the public, key stakeholders, and non-governmental agencies, is a critical point in implementing health-related interventions^39^. It has been successfully used to improve child health, reduce sedentary behaviors, and increase access to healthcare services^40^. Working to enhance the credibility of the tobacco regulatory body among smokers would help in the implementation process; however, it may not increase smoking cessation without effective communication on quitting aids and methods.

### Limitations

This study has several limitations. First, the instruments were self-reported, which increases social desirability bias. Second, the findings may not be generalizable to the general population of Saudi smokers. Third, as this study was cross-sectional, the causality of the credibility of the tobacco control regulatory body and smoking behavior change could not be determined. Moreover, because credibility was not measured longitudinally, it was not possible to detect how rapidly the credibility was formed after tobacco control policy implementations in the country. Finally, to increase the sample size, cigarette tobacco and hookah tobacco were merged into tobacco smoking in general, but were not analyzed separately.

## CONCLUSIONS

This study is a first assessment of the credibility of the tobacco control regulatory body as perceived by smokers in SA, and its association with smoking behavior change. The credibility of the tobacco control regulatory body was positively associated with motivation to change smoking behavior but negatively associated with NRT use. Several approaches can be used to enhance the credibility of the tobacco control regulatory body in SA. Involving the community before, during, and after the implementation process would improve the credibility because it bridges the gap between the authority and the public. Using media to strengthen trust and draw a positive image for the tobacco control regulatory body is another approach to enhance the perceived credibility of smokers. Effective communication with the public is a potential avenue for optimizing tobacco treatment services through tobacco control policies.

## Data Availability

The data supporting this research are available from the authors on reasonable request.

## References

[1] Montazeri Z, Nyiraneza C, El-Katerji H, Little J (2017). Waterpipe smoking and cancer: systematic review and meta-analysis. Tob Control.

[2] Goldklang M, Stockley R (2016). Pathophysiology of Emphysema and Implications. Chronic Obstr Pulm Dis.

[3] Messner B, Bernhard D (2014). Smoking and cardiovascular disease: mechanisms of endothelial dysfunction and early atherogenesis. Arterioscler Thromb Vasc Biol.

[4] World Health Organization Global Adult Tobacco Survey. Fact Sheet: Kingdom of Saudi Arabia.

[5] Hamadeh RR, Borgan SM, Khabsa J, Sibai AM (2021). Tobacco Research in the Eastern Mediterranean Region: A Scoping Review of Published Studies from Seven Countries. J Community Health.

[6] Hassounah MM, Al-Zalabani AH, AlAhmari MD, Murriky AA, Makeen AM, Alanazi AMM (2020). Implementation of Cigarette Plain Packaging: Triadic Reactions of Consumers, State Officials, and Tobacco Companies-The Case of Saudi Arabia. Int J Environ Res Public Health.

[7] Itumalla R, Aldhmadi B (2020). Combating tobacco use in Saudi Arabia: a review of recent initiatives. East Mediterr Health J.

[8] Alotaibi HF, Alsanea NA (2022). Impact of taxation policy on tobacco consumption in Saudi Arabia. Ann Saudi Med.

[9] Aljuaid SO, Alshammari SA, Almarshad FA (2020). Taxation and tobacco plain packaging effect on Saudi smokers quitting intentions in Riyadh city, Saudi Arabia. Saudi Med J.

[10] Goel S, Kar SS, Verma M, Sivanantham P, Naik BN, Gupta D (2021). Evidence on article 5.3 of FCTC (tobacco industry interference in tobacco control activities) in India- a qualitative scoping study. BMC Public Health.

[11] Liberman J (2013). Plainly constitutional: the upholding of plain tobacco packaging by the High Court of Australia. Am J Law Med.

[12] Egbe CO, Bialous SA, Glantz S (2019). Role of stakeholders in Nigeria’s tobacco control journey after the FCTC: lessons for tobacco control advocacy in low-income and middle-income countries. Tob Control.

[13] Egbe CO, Bialous SA, Glantz S (2019). Framework Convention on Tobacco Control Implementation in Nigeria: Lessons for Low- and Middle-Income Countries. Nicotine Tob Res.

[14] Yong HH, Borland R, Siahpush M (2005). Quitting-related beliefs, intentions, and motivations of older smokers in four countries: findings from the International Tobacco Control Policy Evaluation Survey. Addict Behav.

[15] Evans-Polce RJ, Castaldelli-Maia JM, Schomerus G, Evans-Lacko SE (2015). The downside of tobacco control? Smoking and self-stigma: A systematic review. Soc Sci Med.

[16] National Tobacco Control Committee Goals and members of the National Tobacco Control Committee. In Arabic.

[17] Spence AD, Khasawneh M, Allen PB, Addley J (2017). Communication of alcohol and smoking lifestyle advice to the gastroenterological patient. Best Pract Res Clin Gastroenterol.

[18] Guassora AD, Gannik D (2010). Developing and maintaining patients’ trust during general practice consultations: the case of smoking cessation advice. Patient Educ Couns.

[19] Osman A, Kowitt SD, Sheeran P, Jarman KL, Ranney LM, Goldstein AO (2018). Information to Improve Public Perceptions of the Food and Drug Administration (FDA’s) Tobacco Regulatory Role. Int J Environ Res Public Health.

[20] Kowitt S, Sheeran P, Jarman K (2017). Effects of Framing Proximal Benefits of Quitting and Motivation to Quit as a Query on Communications About Tobacco Constituents. Nicotine Tob Res.

[21] Choi K, Hennrikus DJ, Forster JL, Moilanen M (2013). Receipt and redemption of cigarette coupons, perceptions of cigarette companies and smoking cessation. Tob Control.

[22] Saudi Vision 2030 The Quality of Life Program.

[23] Camenga DR, Kong G, Cavallo DA (2014). Alternate tobacco product and drug use among adolescents who use electronic cigarettes, cigarettes only, and never smokers. J Adolesc Health.

[24] Saddleson ML, Kozlowski LT, Giovino GA, Homish GG, Mahoney MC, Goniewicz ML (2016). Assessing 30-day quantity-frequency of U.S. adolescent cigarette smoking as a predictor of adult smoking 14 years later. Drug Alcohol Depend.

[25] Boudreaux ED, Sullivan A, Abar B, Bernstein SL, Ginde AA, Camargo CA (2012). Motivation rulers for smoking cessation: a prospective observational examination of construct and predictive validity. Addict Sci Clin Pract.

[26] Bertholet N, Gaume J, Faouzi M, Gmel G, Daeppen JB (2012). Predictive value of readiness, importance, and confidence in ability to change drinking and smoking. BMC Public Health.

[27] Schmidt AM, Ranney LM, Noar SM, Goldstein AO (2017). Development of the FDA Tobacco Credibility Scale (FDA-TCS). Tob Regul Sci.

[28] Kowitt SD, Schmidt AM, Hannan A, Goldstein AO (2017). Awareness and trust of the FDA and CDC: Results from a national sample of US adults and adolescents. PLoS One.

[29] Elimam M (2021). The Determinations of Public Trust in the Government of Egypt: An Empirical Study.

[30] Kelly PJ, Townsend CJ, Osborne BA (2018). Predicting Intention to Use Nicotine Replacement Therapy in People Attending Residential Treatment for Substance Dependence. J Dual Diagn.

[31] Kim N, McCarthy DE, Loh WY (2019). Predictors of adherence to nicotine replacement therapy: Machine learning evidence that perceived need predicts medication use. Drug Alcohol Depend.

[32] Juliano LM, Brandon TH (2004). Smokers’ expectancies for nicotine replacement therapy vs. cigarettes. Nicotine Tob Res.

[33] Carpenter MJ, Ford ME, Cartmell K, Alberg AJ (2011). Misperceptions of nicotine replacement therapy within racially and ethnically diverse smokers. J Natl Med Assoc.

[34] Rose JE, Behm FM, Drgon T, Johnson C, Uhl GR (2010). Personalized smoking cessation: interactions between nicotine dose, dependence and quit-success genotype score. Mol Med.

[35] Khariwala SS, Rubin N, Stepanov I (2019). “Cold turkey” or pharmacotherapy: Examination of tobacco cessation methods tried among smokers prior to developing head and neck cancer. Head Neck.

[36] Clinical Practice Guideline Treating Tobacco Use and Dependence 2008 Update Panel, Liaisons, and Staff (2008). A clinical practice guideline for treating tobacco use and dependence: 2008 update. A U.S. Public Health Service report. Am J Prev Med.

[37] Smith AL, Carter SM, Chapman S, Dunlop SM, Freeman B (2015). Why do smokers try to quit without medication or counselling? A qualitative study with ex-smokers. BMJ Open.

[38] Lachance C, Frey N (2020). Non-Pharmacological and Pharmacological Interventions for Smoking Cessation Programs in Youth: A Review of Clinical Effectiveness and Guidelines.

[39] Cyril S, Smith BJ, Possamai-Inesedy A, Renzaho AM (2015). Exploring the role of community engagement in improving the health of disadvantaged populations: a systematic review. Glob Health Action.

[40] Ssewanyana D, Abubakar A, van Baar A, Mwangala PN, Newton CR (2018). Perspectives on Underlying Factors for Unhealthy Diet and Sedentary Lifestyle of Adolescents at a Kenyan Coastal Setting. Front Public Health.

